# Extrafollicular IgD+ B cells generate IgE antibody secreting cells in the nasal mucosa

**DOI:** 10.1038/s41385-021-00410-w

**Published:** 2021-05-28

**Authors:** Alessia Corrado, Richard P. Ramonell, Matthew C. Woodruff, Christopher Tipton, Sarah Wise, Joshua Levy, John DelGaudio, Merin E. Kuruvilla, Kelly R. Magliocca, Deepak Tomar, Swetha Garimalla, Christopher D. Scharer, Jeremy M. Boss, Hao Wu, Sanjeev Gumber, Chris Fucile, Greg Gibson, Alexander Rosenberg, Iñaki Sanz, F. Eun-Hyung Lee

**Affiliations:** 1 Division of Pulmonary, Allergy, Critical Care & Sleep Medicine, Department of Medicine, Emory University, Atlanta, GA USA; 2 Division of Rheumatology, Department of Medicine, Emory University, Atlanta, GA USA; 3 Lowance Center for Human Immunology Emory University, Atlanta, GA USA; 4 Department of Otolaryngology, Emory University, Atlanta, GA USA; 5 Department of Pathology and Laboratory Medicine, Emory University, Atlanta, GA USA; 6 School of Biological Sciences, Georgia Institute of Technology, Atlanta, GA USA; 7 Department of Microbiology and Immunology, Emory University, Atlanta, GA USA; 8 Department of Biostatistics and Bioinformatics, Emory University, Atlanta, GA USA; 9 Yerkes National Primate Research Center, Emory University, Atlanta, GA USA; 10 Department of Microbiology and Immunology, Informatics Institute, University of Alabama, Birmingham, AL USA

## Abstract

Increased IgE is a typical feature of allergic rhinitis. Local class-switch recombination has been intimated but B cell precursors and mechanisms remain elusive. Here we describe the dynamics underlying the generation of IgE-antibody secreting cells (ASC) in human nasal polyps (NP), mucosal tissues rich in ASC without germinal centers (GC). Using *V*_H_ next generation sequencing, we identified an extrafollicular (EF) mucosal IgD+ naïve-like intermediate B cell population with high connectivity to the mucosal IgE ASC. Mucosal IgD+ B cells, express germline epsilon transcripts and predominantly co-express IgM. However, a small but significant fraction co-express IgG or IgA instead which also show connectivity to ASC IgE. Phenotypically, NP IgD+ B cells display an activated profile and molecular evidence of BCR engagement. Transcriptionally, mucosal IgD+ B cells reveal an intermediate profile between naïve B cells and ASC. Single cell IgE ASC analysis demonstrates lower mutational frequencies relative to IgG, IgA, and IgD ASC consistent with IgE ASC derivation from mucosal IgD+ B cell with low mutational load. In conclusion, we describe a novel mechanism of GC-independent, extrafollicular IgE ASC formation at the nasal mucosa whereby activated IgD+ naïve B cells locally undergo direct and indirect (through IgG and IgA), IgE class switch.

## Introduction

IgE antibodies play a key role in the pathogenesis of severe allergic disease and of late, have been implicated in other airway diseases unrelated to atopy. IgE is produced by IgE antibody secreting cells (ASC) that arise from terminally differentiated B cells in the presence of Th2 cytokines IL-4 and IL-13. Among the immunoglobulin isotypes, IgE has the lowest abundance in the serum and the shortest half-life^1^ suggesting that ongoing IgE secretion is necessary to maintain tissue or serum levels in disease. More intriguingly, mucosal and not serum IgE levels correlate with disease severity in allergic patients^2^ illustrating that local ASC in tissues are responsible for disease pathogenesis.

The generation of plasma cells requires naïve B (nB) cell activation via germinal center (GC) reactions^3^ to undergo affinity maturation by somatic hypermutation as they differentiate into in high affinity ASC or memory B cells^4^. Alternatively, nB cells can also undergo extrafollicular (EF) reactions outside the GC and directly differentiate into ASC as described initially in mice models of Ehrlichia muris, Salmonella enterica serovar Typhimurium, and Borrelia burgdorferi^5–15^ and more recently in humans in autoimmunity and primary viral infections such as SARS-CoV-2^16–19^. Reports have shown that a primary immune response can initiate class switching prior to GC formation^20–22^, and more recently, that class switching predominantly takes place at the T-B border and infrequently in the GC^23^. Additionally, studies in lymphotoxin or tumor necrosis factor mutant mice which lack normal lymphoid architecture also demonstrate mutation and affinity maturation can occur in EF B cell responses^24^. Whether the majority of mucosal IgE ASC differentiate primarily in lymph nodes EF or GC reactions or predominate in the local EF mucosa sites has remained elusive.

Due to the rare nature of human IgE ASC, understanding their origin has been difficult to study; thus, mouse models have proven invaluable. Unfortunately, the parasitic models of IgE GFP reporter mice have been contradictory. One study describes a considerable fraction of IgE^+^ GC B cells survive and differentiate into IgE memory B cells (mB) and IgE ASC according to the classical IgG_1_^+^ GC cell program^25^. They also suggest that IgE ASC can reside in the bone marrow, although less efficiently than the IgG_1_ ASC, to ensure a constant serum IgE level^25^. Others show that IgE^+^ B cells in the GC are transient with a high propensity to upregulate BLIMP-1 and rapidly differentiate into ASC, thereby making IgE^+^ B cells extremely rare. Thus IgE mB cell are controversial possibly due to their rapid transition into an ASC^26–28^. In all, there may be several mechanisms of IgE ASC generation even in mouse models.

The first of two mechanisms of IgE ASC class-switch recombination (CSR) is direct class switching from IgM to IgE while the second mechanism is sequential switching through intermediaries such as IgG or IgA. In mice, it has been well-described that GC IgG1^+^ B cell intermediates can class switch to IgE ASC outside the GC^27^. This model was further validated by impairment of high affinity IgE production in IgG_1_-deficient mice^29^. Interestingly, these cognate IgE ASC are more mutated than the IgM/IgD B cell precursors^30^ suggesting two-step class switching that results in relatively higher mutation rates and affinities compared to IgE from direct class switching^28,29^. One recent human study described that direct IgM/IgD to IgE switching occurs at a lower frequency compared to sequential IgG or IgA1 to IgE class switching^30^. Additionally, several studies also show evidence of local class switching with increased RAG1, RAG2, and AID expression together with epsilon-germline transcripts from total nasal biopsies of atopic patients during the allergy season^31–33^. Despite evidence of local class switching, whether the IgE ASC originated from EF or GC origins were not clearly elucidated.

Based on improved knowledge of human B cell populations derived from recent studies, we sought to characterize the B cell precursors of IgE ASC through a combination of phenotypic, BCR repertoire and molecular analyses. Our experimental model was based on the hypothesis that the study of nasal polyps (NP), would provide a rich environment for ongoing IgE ASC differentiation from different cellular precursors in the absence of GC. In keeping with this model, we identified a novel human EF mucosal B cell population (CD19^+^IgD^+^CD27^−^) as the origin of mucosal IgE ASC. This population shared many of the phenotypic and transcriptional features of activated naive B cells recently shown in the EF response of patients with active autoimmune disease and severe SARS-CoV-2 infection^16–19^. These mucosal IgD+ B cells are characterized by: (1) a high degree of clonal connectivity with IgE ASC, (2) an activated phenotype (CD11c+CD86+CD71+Nur77+CD21−), (3) a progressive transcriptome between resting blood naïve B cells and NP ASC, and (4) the highest level of germline epsilon transcripts. Notably, the majority of the IgD+ NP B cells expressed IgM sequences with low mutation rates, a profile consistent with activated naïve B cells. However, IgD+ NP B cells also contained a smaller fraction of cells with very high rates of somatic hypermutation previously demonstrated in class-switched IgD-only B cells (IgD+IgM−; Cδ-CS), reported in the literature within either GC, memory and ASC populations^34^. Interestingly, the analysis of single cell IgE ASC demonstrated lower mutation relative to ASC of other isotypes including IgG, IgA, or the highly mutated IgD ASC. In summary, human mucosal IgE ASC originate from an EF mucosal activated IgD+ B cell subset in patients with atopic and non-atopic airways disease.

## Results

### Elevated pollen counts do not increase peripheral blood IgE ASC frequencies in allergic adults

**Fig. 1:**
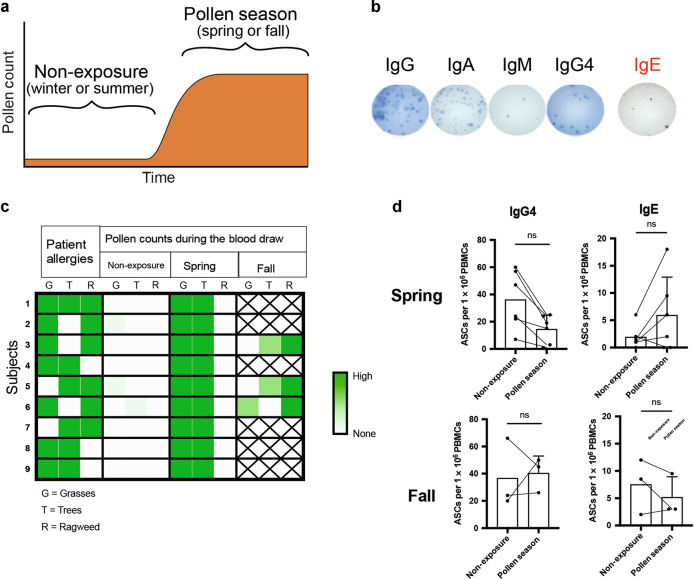
Total circulating IgG, IgA, IgM, IgG4 and IgE secreting cells. **a** Schematic of blood draws prior to (pre-exposure: PE) and during periods of high pollen counts (pollen season: PS) in Atlanta, GA. **b** Representative IgG, IgA, IgM, IgG_4_ and IgE Elispots from PBMC of a patient with a high IgE serum titer. Input PBMC numbers/well: 333 K. **c** Heat map of 9 atopic subjects and confirmed allergy to grasses (G), trees (T), and ragweed (R) and the pollen counts during the non-season, Spring when grass and tree pollens were high and the fall when the ragweed counts were high in Atlanta when the corresponding blood samples were drawn. **d** Frequencies of IgG_4_ and IgE ASC Elispots in PBMC from the 9 atopic subjects prior to the allergy season (non-exposure: NE) and during the pollen season (PS) in the spring and fall.

In response to vaccination or infection, antigen-specific ASC increase in the blood a few days after as they leave the lymph node (LN) to reside in target tissues, spleen and bone marrow (BM)^35,36^. By analogy, we hypothesized a rise of blood IgE ASC when allergic patients re-encounter allergens during the pollen season. We recruited 74 atopic patients with history of allergic rhinitis and/or asthma with high serum IgE levels (>180 IU/mL) and positive skin prick testing or specific serum IgE >0.35kU/L for common aeroallergens and 21 healthy non-atopic adults (Supplementary Table 1). Patients on oral corticosteroids or subcutaneous or sublingual immunotherapy were excluded. Blood samples were analyzed during non-aeroallergen exposure (NE) (winter or summer) and during the high pollen season (PS) (spring or fall) (Fig. 1a). The pollen season was defined by periods when aeroallergen counts were elevated in Atlanta, Georgia, as indicated by the Atlanta, Allergy & Asthma’s Pollen Counting Station certified by the National Allergy Bureau (http://www.atlantaallergy.com/). Nearly all patients were symptomatic during periods of the high pollen counts.

Frequencies of circulating IgG, IgA, IgM, IgG_4_ and IgE ASC in patients with allergies were quantified by Elispots (Fig. 1b). During the non-aeroallergen season (NE), frequencies of non-IgE (IgG, IgA and IgM) ASC in PBL were similar in both allergic patients and healthy donor controls (HD). In the atopic patients, the non-IgE ASC frequencies before (NE) and during the high aeroallergen seasons (PS) were also similar in blood (Supplementary Fig. 1a). In contrast, IgE and IgG_4_ ASC, which can concomitantly rise together^37^, were slightly increased in the NE or PS in allergic patients compared to HD. (IgG_4_: HD vs NE *p* = 0.0427, HD vs PS *p* = 0.0088 and IgE: HD vs NE *p* < 0.0001, HD vs AS *p* < 0.0001, *t*-test) (Supplementary Fig. 1a). However, among the atopic patients, there was no difference between IgE and IgG_4_ ASC frequencies in PBL in NE (winter and summer) and PS (spring and fall).

To insure we only evaluated atopic patients who had documented allergies to the circulating aeroallergen, we selected a group of atopic subjects with positive skin prick or IgE blood tests specific for grasses, trees, and/or ragweed and collected the blood during the non-allergen season, and during pollen season (spring when the grasses and tree pollens were high and the fall when the ragweed counts were elevated in Atlanta (Fig. 1c). Again, total IgG_4_ and IgE ASC frequencies in the blood showed no difference when there were no or low pollen counts (NE) and when the pollen counts were high (PS) either in the spring or fall (Fig. 1d). We also found no direct correlations with serum IgE titers and PBL IgE ASC frequencies (Supplementary Fig. 1b). In conclusion, circulating IgE ASC do not increase during the high pollen season demonstrating IgE ASC released from LNs may not play a major role in allergy symptoms. This data continues to support the role of local IgE ASC formation as others have shown^31–33^, and mechanisms of IgE ASC generation in allergic diseases differ from IgG and IgA ASC formation after vaccination and infection.

### Total ASC and IgE ASC are highly enriched in nasal polyps in the absence of GC

**Fig. 2:**
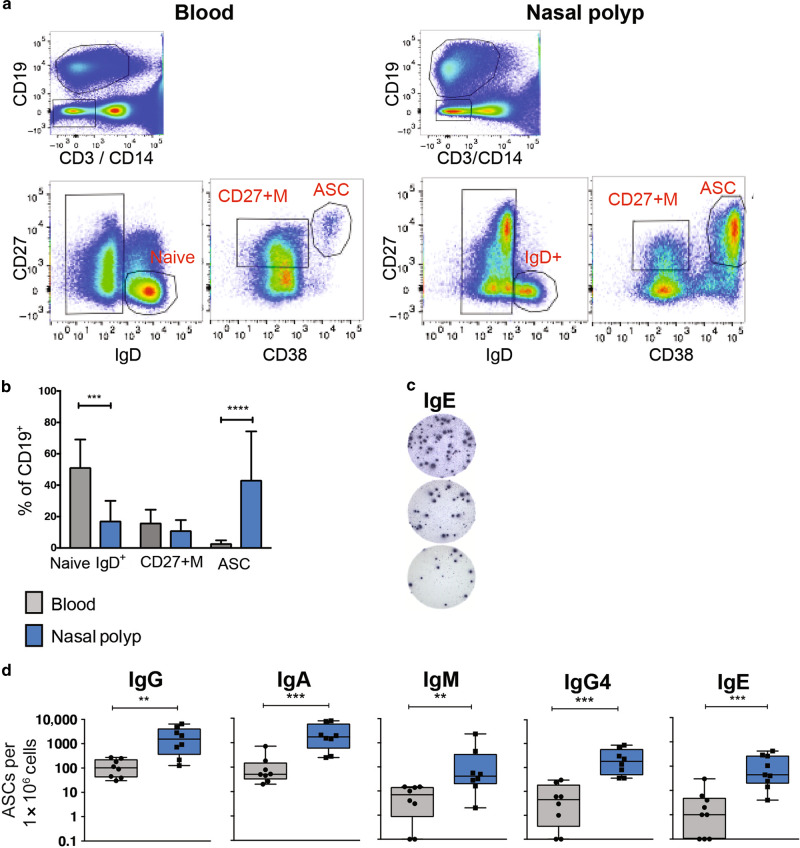
Comparison of B cell subsets and ASC in peripheral blood (PBL) and nasal polyp (NP). **a** Flow panel of B cell subsets in the PBL (left) and in NP (right): naïve or IgD+ B cell: CD19+IgD+CD27−, CD27+ memory B cell (CD27+M): CD19+IgD−CD27+CD38−, and antibody secreting cells (ASC): CD19+IgD−CD27+CD38++. **b** Frequency of B cell subsets (Naïve or mucosal IgD+ B cell, CD27+M B cell and ASC) of total CD19+ cells in the blood or nasal polyp. PBMC in gray bars, NP in blue bars. Mean + SD of 12 nasal polyp and matching blood samples is shown. **c** Representative IgE Elispots from a nasal polyp. Serial dilution of NP cells: 250,000, 83,333, and 27,777 cells per well. **d** Frequencies of total IgG, IgA, IgM, IgG_4_, and IgE ASC measured by Elispots, performed on 9 matching nasal polyp and blood samples. Mean + SEM are shown. (*p* value * 0.05, ** 0.01, *** 0.001, **** 0.0001, Mann–Whitney).

We then analyzed cells from the NP of patients with aeroallergen sensitization. NP are non-cancerous lesions arising from the nasal mucosa or sinuses and frequently associated with rhinitis and asthma^38^. We collected matching NP and blood samples from patients undergoing surgery with chronic rhinosinusitis (CRS), allergic fungal sinusitis (AFS) or aspirin exacerbated respiratory disease (AERD) and measured B cell subsets by flow cytometry (Fig. 2a). We found significantly decreased frequencies of IgD+ B cells (CD19^+^IgD^+^CD27^-^) and higher frequencies of total ASC (CD19^+^IgD^−^CD38^hi^CD27^hi^) in the NP compared to PBL (*p* value 0.0003 and <0.0001 respectively, Mann–Whitney test). Of further note, NP samples were also enriched for IgD−CD27− double negative B cells, an EF population we previously described in patients with active systemic lupus erythemathosus (SLE)^16^. In contrast, there were no differences in numbers of CD27+ memory (CD27+M) cells (CD19^+^IgD^−^CD38^−^CD27^+^) between blood and NP (Fig. 2b).

Consistent with the flow analysis, Elispots assays confirmed in NP the presence of higher numbers of functional ASC spontaneously secreting IgG, IgA, IgM and IgG_4_ as well as IgE antibodies (Fig. 2c, d). Moreover, the IgE ASC frequency was much higher in NP tissues relative to other lymphoid organs with abundant follicles and germinal centers (GC). In contrast, no significant differences in non-IgE (IgG, IgA, IgM and IgG_4_) ASC were found between NP and lymphoid tissue (Supplementary Fig. 2). These findings are in keeping with the proposed IL-21-mediated inhibition of IgE class switching in lymphoid GC^39–42^. Finally, we identified similar frequencies of IgG, IgA, and IgM ASC in the bone marrow (BM) of atopic patients compared to NP and other lymphoid tissues. In contrast, BM IgE ASC were higher than in the blood and lymphoid tissues albeit lower than in NP. In summary, NP contain the highest frequency of IgE ASC relative to any other tissue including the BM (Supplementary Fig. 2).

**Fig. 3:**
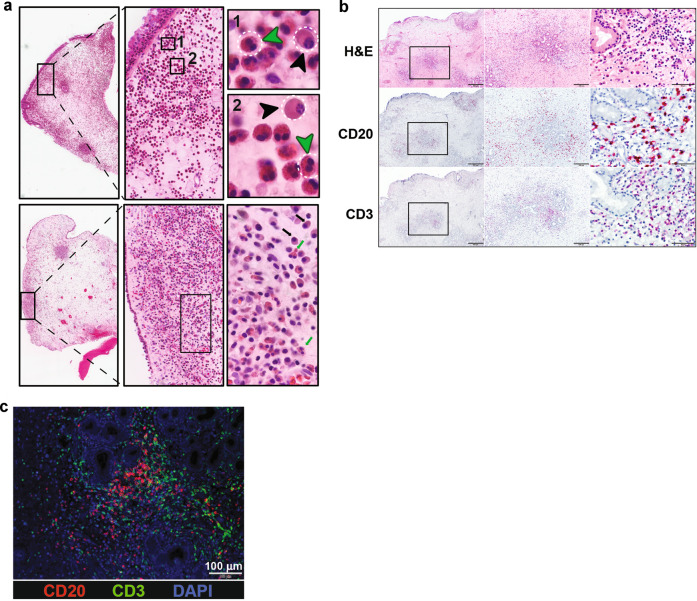
Histology of Nasal Polyps. **a** Hematoxylin and Eosin staining of two representative nasal polyps, 30×, 200×, 1000× magnifications are shown. Black squares highlight clusters of plasma cells and lymphocytes. In the 1000× magnification, black and green arrows indicate plasma cells and eosinophils, respectively. **b** No GC-like structures identified in nasal polyp tissues. Top panel: H&E, CD20, and CD3 staining 30×, 100×, 600×. **c** Immunofluorescent staining of nasal polyp, blue: DAPI, red:anti-CD20- PE, green:anti-CD3-FITC.

NP are frequently a result of type 2 inflammation and contain lymphoid (T and B cells) and stromal cells, eosinophils, neutrophils, and mast cells, together with an abundance of ASC^43^. We stained seven NP samples with hematoxylin & eosin, and anti-CD20 and anti-CD3 for lymphoid follicles (Fig. 3 These studies established that, while numerous ASC, eosinophils and neutrophils could be readily identified in NP, these tissues were completely devoid of GC structures (Fig. 3a), even in lymphocyte-dense areas with high concentration of B and T cells (Fig. 3b, c). These results are similar to what others had shown^44^.

### Mucosal IgD+ B cells are a major source of IgE ASC in NP

**Fig. 4:**
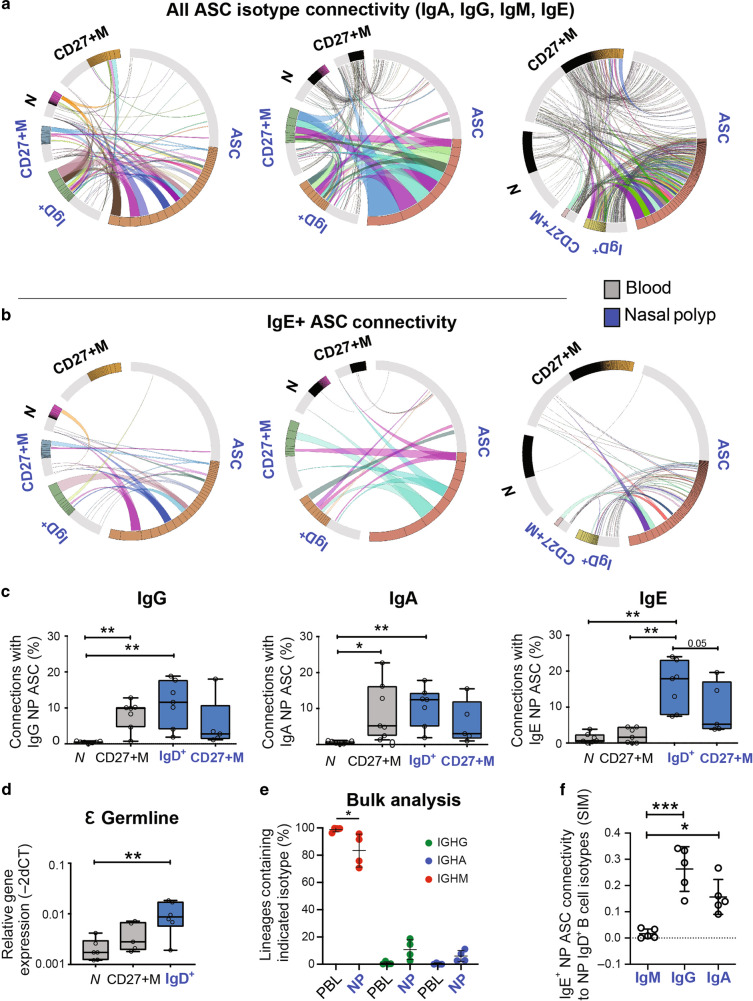
Connectivity of nasal polyp (NP) antibody secreting cells (ASC) with matching NP and blood B cell subsets using VH repertoire sequencing. **a** Circos plots of 3 representative NP and blood (PBL) of 7 total showing clonal interrelatedness: total NP ASC (IgG, IgA, IgM, IgE isotypes) connections with NP IgD+ (IgD+) and NP CD27+ Memory Cells (CD27+M) (annotated in blue) and PBL Naïve (N) and PBL CD27+M (annotated in black). Clonal sizes displayed in the outer track with highlighted portions of the group track indicating the (top 50% of sequences) D50. The inner space illustrates connections between the NP ASC and B cell subsets in NP and PBL. Colored connected lines represent relationships between clonotypes that consist of more than 50 sequences, and gray connected lines denote relationships of clonotypes with fewer than 50 sequences. **b** Circos plots of the same 3 representative NP of 7 showed displaying only NP ASC IgE isotype connectivity. **c** Percentages of IgG (left), IgA (middle) and IgE (right) NP ASC clonal lineages shared among Naïve and CD27+ M B cells from PBL (in black) and matching NP (in blue). Total of 7 NP and matching PBL samples. Note NP CD27+ mB cells that were sequenced only for 5 samples, and Paired *t*-test (two tailed) was applied (*p* value * 0.05, ** 0.01, *** 0.001, **** 0.0001). The IgE ASC and NP IgD+ B cell connectivity differed significantly from IgE ASC and other PBL subsets (PBL CD27+ m vs NP IgD+ B cells *p* = 0.0043, the PBL nB cells vs. NP IgD+ B cells *p* = 0.0016, paired *t*-test two tailed) showing that mucosal IgD+ B cells relevant to the local allergic response had to be selected and represented the main source of tissue IgE ASC. **d** Relative expression of Epsilon germline transcripts measured using SYBR Green Real Time PCR and normalized to β-actin from 6 different matching NP IgD+ B cells and compared to PBL nB cells. Mann–Whitney (two tailed) was applied (*p* value * 0.05, ** 0.01, *** 0.001, **** 0.0001). **e** Next generation sequencing performed in bulk to determine the isotype distribution of NP IgD+ (blue) and PBL naïve (black) B cell subsets from 4 additional individuals. The sequencing was performed utilizing combined IgM, IgG, and IgA primers. IgG isotypes (green), IgA isotypes (purple), IgM isotypes (red). **f** Fraction of NP IgE ASC connectivity related to isotypes IgG, IgA, and IgM in NP IgD+ B cells (7 NP samples).

To investigate B cell origins of the NP IgE ASC, we performed deep BCR VH repertoire sequencing from B cell subsets from the PBL and NP of seven matched adults. ASC, IgD+ CD27−, and CD27+ mB cells from the NP as well as blood naïve and CD27+ mB cells were sorted from each compartment and sequenced as previously described^18,45^. Clonal relationships between NP ASC and distinct B cell subsets were analyzed and the numbers of cells, sequences and lineages (clones) are listed in Supplementary Table 2. Representative circos plots from 3 different subjects, displaying clonal connectivity between NP ASC of all isotypes (IgA, IgG, IgM and IgE combined) and B cell subsets from blood or NP tissues are shown in Fig. 4a. Circos plots from the same sequencing data but displaying only the connections between IgE-specific NP ASC and other B cell subsets, are shown in Fig. 4b.

The repertoire analysis contributed several insights of significance. First, NP ASC (all isotypes) showed clear-cut connectivity to the NP IgD+ and CD27+ mB cells (Fig. 4a) with a vast majority of which were shared with NP IgD+ cells. Second, the NP IgD+ B cells displayed the largest fraction of shared clonal lineages with IgE ASC with significant statistical differenceswith NP mB cell (Fig. 4b). In contrast, there was minimal connectivity between IgE ASC and any PBL B cell subsets (Fig. 4c). For IgG or IgA ASC, there were ample connections between the NP IgD+ cells as well as the CD27+ m B cells from the blood or mucosa suggesting multiple B cell sources. Collectively, the results indicate that NP IgG and IgA ASC can originate from both blood and mucosal B cell subsets whereas IgE ASC originate predominately from NP IgD+ B cells.

To further confirm NP IgD+ B cells as potential precursors of IgE ASC, we performed RT-PCR of the ε-germline transcripts since class-switch recombination to IgE results in production of ε-sterile germline transcripts of the CH gene^46,47^. As expected, the NP IgD+ B cells expressed significantly more ε-germline transcripts than PBL nB and CD27+ B cells from the same subjects (*p* value 0.0079, Mann–Whitney test, two tailed) (Fig. 4d).

A fraction of NP IgD+ B cells had already undergone class switch by bulk NGS in the NP IgD+ B cell populations in an additional 4 patients using NP IgD+ B cells and PBL nB cells for each isotype using a single multiplex PCR with a mix of IgG, IgA, and IgM constant region reverse primers (Fig. 4e). This approach allowed for precise quantification of the isotypes expressed. Post-sort purity which was 97–99% of the IgD+ B cells in the NP, and as expected nearly all resting PBL nB cells contained only IgM transcripts. In contrast, the NP IgD+ B cells encompassed mostly IgM but also few IgG and IgA sequences (83%, 11%, and 6%, respectively). The PBL nB cell fractions contained more than 99% IgM.

To address the question of direct vs. sequential IgE class switching, we examined the relatedness of IgE ASC clonality with NP IgD+ B cells isotypes. IgE ASC connectivity with NP IgD+ B cells of IgG isotype (SIM index of 0.28) or IgA isotype (SIM index of 0.15) and IgM (SIM index of 0.01). These results support a model of sequential IgE class switching due to abundance of these IgG and IgA intermediates (Fig. 4f) similar to what has been reported in other mouse and human studies^27,30,33,48,49^.

### Characterization of the NP (naïve) IgD+ B cell subsets

**Fig. 5:**
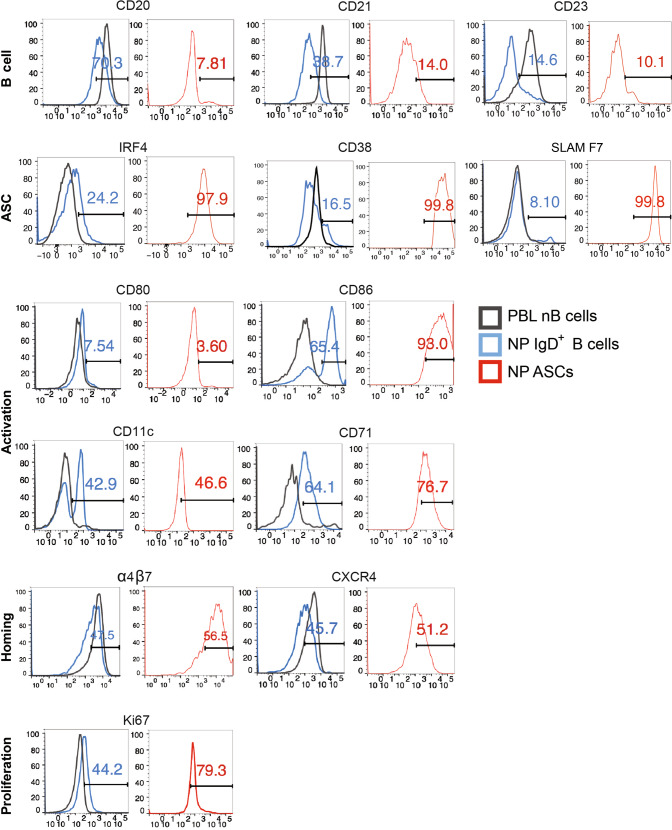
Immunophenotypic characterization of matching PBL and NP from 16 matched PBL and NP were used for flow phenotyping. Representative overlapping histograms from PBL nB cells (CD19^+^IgD^+^CD27^−^) (black), NP IgD+ B cells (CD19^+^IgD^+^CD27^−^) (blue) and NP ASC (CD19^lo^CD27^hi^CD38^hi^) (red) of the following surface marker expression: CD20, CD21, CD23, CD38, CD80, CD86, CD11c, CD71, α4β7, CXCR4, and SLAMF7 and intracellular expression of IRF4, Ki-67. The number of samples analyzed for each marker and the mean MFI are shown in Supplementary Table 3.

While the IgD+CD27− fraction of human peripheral blood B cells is generally considered a naïve population, our studies show significant heterogeneity within this compartment, a feature more readily documented in active SLE patients and primary severe SARS-CoV-2 infection with enhanced EF responses^16–19,50^. In patients with active SLE, IgD+CD27− cells are highly enriched in an activated fraction were termed activated Naïve B cells (aN). These cells represent the first step of naïve B cell differentiation into EF ASC through an IgD−CD27− DN intermediate comprised by a large majority of isotype switched cells expressing significant levels of somatic hypermutation albeit of substantially lower magnitude than isotype switched CD27+ memory cells. Consistent with these observations, flow cytometry analysis demonstrated an activated phenotype of NP IgD+CD27− B cells (CD11c+CD86+CD71+CD21−; Fig. 5).

The NP IgD+ B cells have increased markers of activation as well as partial loss of typical B cell surface markers, and upregulation of ASC receptors (Fig. 5). Using multi-parameter flow cytometry, we show that CD20, a trans-membrane protein expressed on mature B cells is highly expressed on all PBL nB cells, whereas it is typically downregulated with initiation of plasma cell differentiation^51^. Accordingly, the NP nB cells experienced partial loss of CD20 (70% of expression compared to 100% in PBL naïve) (Fig. 5, Supplementary Table 3). In addition, the NP IgD+ B cells showed 50–60% co-expression with IgM and IgD as others had previously shown^52^. CD21 and CD23 are normally expressed during the immature and mature stages of B cell development^53,54^ and oftentimes downregulated with activation. The NP IgD+ B cells displayed decreased surface expression of CD21 (40%) and CD23 (34%) compared to 90% and 80% respectively on PBL nB cells (Fig. 5, Supplementary Table 3). We also observed increased expression of plasma cell markers on these cells such as intracellular IRF-4, surface SLAMF7, and CD38 (which is transcribed during plasma cell differentiation^55,56^) (Fig. 5, Supplementary Table 3).

Also of interest was Ki-67^+^, which was 22%, (range 9–44%) showing that a quarter of the NP IgD+ B cells were proliferating (Fig. 5, Supplementary Table 3). There was also decreased expression of CXCR4 and α4β7 on NP IgD+ B cells compared to PBL nB cells implicating the loss of homing markers upon arrival to mucosal sites. In conclusion, NP IgD+ B cells have increased markers of proliferation, activation, and plasma cell differentiation consistent with an intermediary or transitory population between resting PBL nB cells and fully differentiated mucosal ASC.

**Fig. 6:**
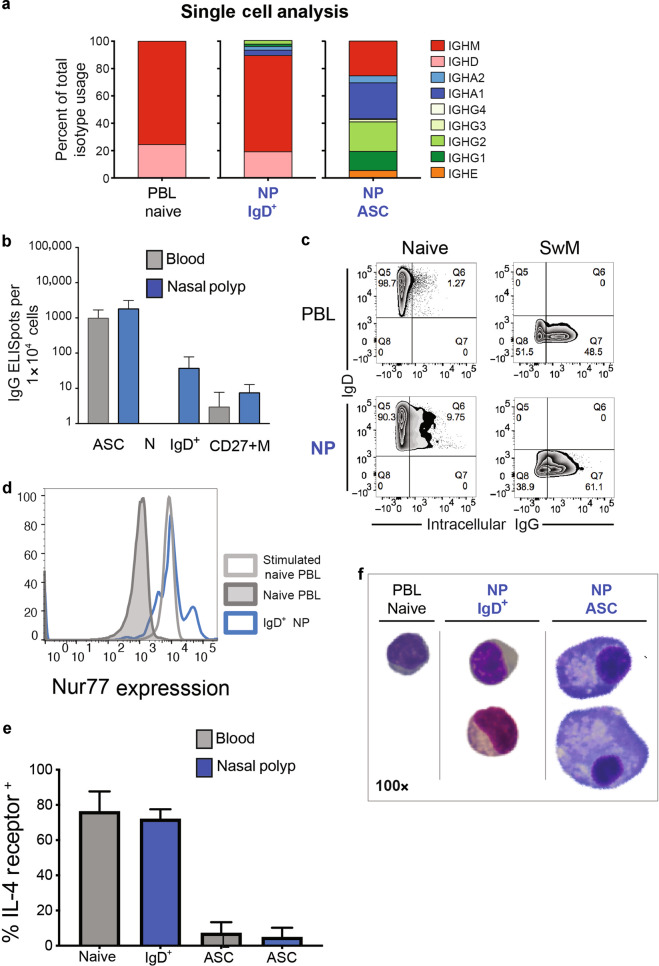
Characterization of NP nB cells. **a** 10× single cell VDJ immune profiling analysis of 798 PBL naïve B cells (left), 219 NP IgD+ B cell (middle), and 210 NP ASC (right) from one allergic subject with NP and matching PBL. 10× primers for IgM, IgA_1&2_, IgG_1-4_, IgE, and IgD utilized. **b **Spontaneous IgG Elispots from 3 additional matching NP & PBL: ASC, IgD+ or naïve and CD27+ M B cells were sorted and analyzed for the production of IgG. **c** Flow staining for surface IgD and intracellular IgG of IgD+ or naïve and CD27+ M B cells from a subject with matching NP & PBL (representative of *N* = 3 experiments). **d** Increased intracellular Nur77 expression in NP IgD+ B cells (blue) compared to PBL nB cells (solid gray) and anti-IgM stimulated PBL nB cell as positive control (open gray). Representative of *N* = 5 experiments. **e** Percentage of IL-4 receptor expression in naïve B cells and ASC from PBL (*N* = 7) (gray) and NP IgD+ B cell and ASC (*N* = 2) (blue). **f** Morphology of PBL naïve B cells, NP IgD+ B cells, and NP ASC (*N* = 2) Cytospin of FAC sorted populations by Wright-Geimsa (100× magnification).

A rigorous quantification of the frequency of isotype switched cells within the bulk NP IgD+ B cells and PBL nB cells was obtained for single cell (sc) VDJ profiling on the 10× Genomics platform from sorted IgD+ B cells from each tissue site and NP ASC (Fig. 6a). Again, PBL nB cells expressed only IgD and IgM, whereas NP ASC showed IgD and IgM as well as all switched isotype sequences IgG, IgA, and IgE which could be discriminated by subclasses IgA_1&2_ and IgG_1,2,3,&4._ Similar to the bulk sequencing, we found that NP IgD+ B cells contained several isotype sequences, in particular IgD, IgM, IgA_1_ and IgA_2_ along with IgG_1_ and IgG_2_. No IgG_3_, IgG_4,_ or IgE sequences were identified (Fig. 6a and Supplementary Fig. 2b). The NP IgD+ B cell frequencies of class-switched sequences in single cell analysis were very similar to percentages from the bulk V_H_ repertoires.

A small fraction of NP IgD+ B cells were able to secrete immunoglobulins spontaneously. B cells and ASC from matching NP and PBL were FACS sorted and IgG Elispots were performed. Of the total NP IgD+ B cells, 0.46% while no PBL nB cells secreted IgG. In comparison, 24.3% of total NP ASC spontaneously secreted IgG (Fig. 6b).

To validate the co-expression of IgG in a small fraction of NP IgD+ B cells expressing IgG transcripts, and to rule out contamination with IgG+ B cells or ASC of the sorted cells used for sequencing, we further assessed IgG expression by intracellular IgG flow cytometry. From three matching PBL and NP samples, NP surface IgD+ B cells compared to background (range 1.2–1.4) in PBL nB cells (Fig. 6c). IgG surface staining showed that 8% NP IgD+ B cells were positive for both surface IgD & intracellular IgG and 13% for both surface IgD & intracellular IgA (Supplementary Fig. 3a). These data conclusively show that a fraction of mucosal IgD+ B cells have initiated class switch and express intracellular IgG or IgA by protein and transcript levels.

Testing whether NP IgD+ B cells have undergone B cell receptor engagement, NP B cells were stained for nuclear expression of Nur77, a marker of antigen receptor binding^57,58^. Cells from five new additional matching NP and PBL samples were intracellularly stained with anti-Nur77 antibodies. PBL nB cells stimulated with anti-IgM were positive controls. A large fraction of unstimulated NP IgD+ B cells showed Nur77 expression (average 46% (25–84%)) while only 1.5 % (0.5–2.4%) of resting naïve B cells were positive (Fig. 6d). These results demonstrate that oftentimes more than half of NP IgD+ B cells are antigen-experienced.

IL-4 is essential for IgE and IgG_4_ class switching; therefore, we evaluated the IL-4 receptor (IL-4R) expression on PBL nB cells and ASC (*N* = 7). Nearly 80% of nB cells and NP IgD+ B cells were positive for IL-4R expression while less than 10% were positive on circulating ASC (Fig. 6e). High IL-4R expression on PBL nB cells and NP IgD+ B cells suggest that these cells are readily poised to differentiate to IgE ASC.

A fraction of the NP naïve B cells were larger in size than resting blood naïve B cells but smaller than NP ASC (Fig. 6f) suggesting B cell engagement and activation, and some displayed a plasma cell-like morphology as evidenced by size, prominent cytoplasm, and a smaller nucleus compared to the conventional naïve cells in the blood. Mean forward scatter signal intensity was slightly higher in NP naïve B cells (70,724 MFI) compared to resting blood naïve B cells (64,560 MFI) from the same subject (*p* value 0.0108, paired *t-*test, two tailed) (Supplementary Fig. 3c).

### NP IgD+ B cells can be mutated

**Fig. 7:**
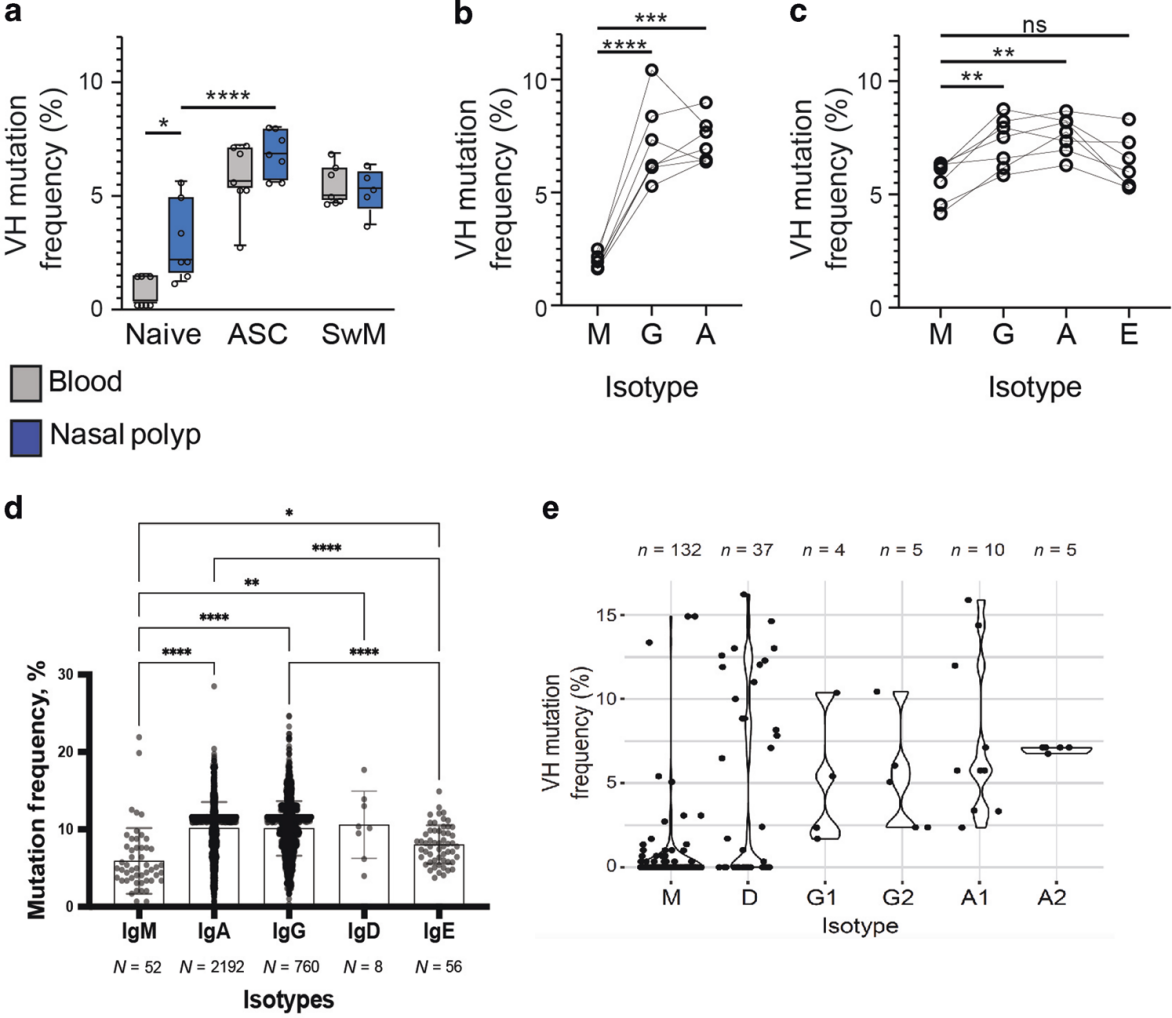
Characteristics of PBL nB cells, NP IgD+ B cells, and NP ASC isotypes and mutation frequencies. **a** Comparison of averaged median lineage mutation frequencies (%) between blood (gray) and NP (blue) resident B cells from indicated populations. Each point represents one individual. **b** Averaged median lineage mutation frequencies of NP IgD+ B cells by isotype (IgM, IgG, and IgA). Each point represents one individual with lines connecting individuals. **c** Averaged median lineage mutation frequencies of NP ASCs by isotype (IgM, IgG, IgA, and IgE). Each point represents one individual with lines connecting individuals. **d** Heavy chain mutation frequencies of single cell IgM, IgG, IgA, IgD, and IgE NP-ASCs. (*N* = 2) Each data point represents a single NP-ASC consensus heavy chain. T-testing (two tailed) *p < 0.05, **p < 0.01, ***p < 0.001, ****p < 0.0001. **e** Heavy chain mutation frequencies of single cell NP IgD+ B cells from Fig. 6b. Isotypes IgM, IgD, IgG1, IgG2, IgA, IgA2 shown. Single point represents a single cell.

Direct or sequential IgE class switching would likely lead to lower mutation rates. Bulk mutation frequencies in NP IgD+ B cells were slightly higher than PBL naïve B cells despite similarly high mutation frequencies in ASC and CD27+ mB cells in blood or NP (Fig. 7a). The reason for the higher mutation frequencies in the NP IgD+ were due to class-switched IgG & IgA compared to unswitched IgM (Fig. 7b). Bulk IgE ASC mutation frequencies in the NP ASC trended lower compared to IgG & IgA but were not statistically significant (Fig. 7c). Extending this analysis to single cells by 10×, we sorted ASC from two NP samples and performed VDJ sequencing and analyzed 4018 single ASC of the following isotypes: 52 IgM, 2192 IgA, 1760 IgG and 8 IgD, and 56 IgE. Of the ASC, IgM had the lowest average mutation frequency followed by IgE and then IgG & IgA. Most interesting was that IgD ASC had the highest average mutation rates although not statistically significant from IgG and IgA probably due to the rarity of these cells (Fig. 7d). To evaluate the mutation frequencies of NP IgD+ cells, we show the mutation frequencies of the NP IgD+ B cells by isotype and subclasses from Fig. 6b. We show that the NP IgD+ cells are a heterogenous population. Similar to the mutation frequencies of the bulk sequencing, IgM was very low, with higher levels in IgA_1,2_ and IgG_1,2_ (Fig. 7e). For IgD+/intracellular IgD+ cells, most had very low mutated frequencies similar to the IgM fraction. However, there was a very small fraction of the intracellular IgD+ sequences that were highly mutated similar to class-switched intracellular IgG and IgA. Due to the higher mutation frequencies in the NP IgD+/intracellular IgD+ B cells compared to the IgE ASC, it is not likely that they are a novel non-canonical IgD to IgE. More likely, these highly mutated IgD+/IgD+ NP B cells are precursors to the highly mutated IgD ASC in the mucosa.

### NP IgD+ B cells have an intermediate transcriptomic phenotype between PBL nB cells and ASC

**Fig. 8:**
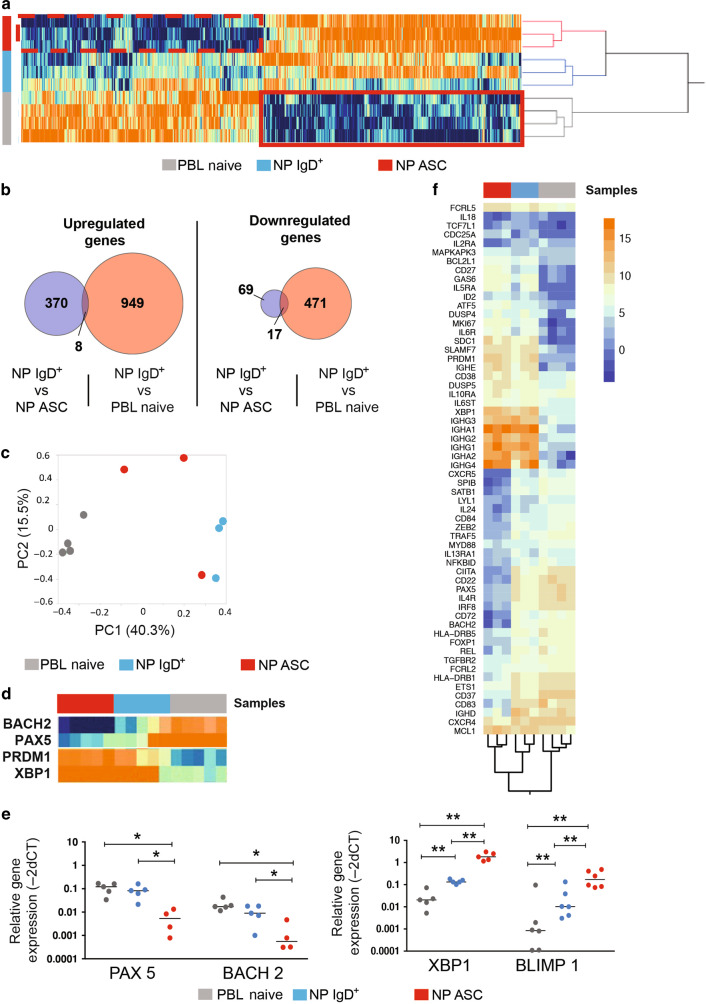
RNAseq analysis of NP ASC, NP IgD+ B cells, and PBL nB cells. **a** Hierarchical clustering of the NP ASC (red) (*N* = 3), NP IgD+ B cells (blue) (*N* = 3), and PBL nB cells (gray) (*N* = 4). **b** DEG between NP IgD+ B cell vs NP ASC and NP IgD+ vs PBL nB cell that were upregulated (left) and downregulated (right). **c** PCA analysis of NP ASC (red), NP IgD+ B cells (blue), and PBL nB cells (gray). **d** Relative expression, measured by SYBR Green RT PCR and normalized using Beta actin, of PAX5, BACH2, XBP1 and BLIMP1 in FAC sorted NP ASC (red), NP IgD+ B cell (blue), and PBL nB cell (gray). **e** Quantitative expression of PAX5, BACH2, XBP1 and BLIMP1 in 4 additional individuals with matching NP and PBL samples. **f** supervised analysis of selected DEG. ASC antibody producing cells, BM bone marrow, EF extrafollicular, GC germinal center, mB memory B cells, NP nasal polyp, nB naïve B cells, PBL peripheral blood, PCA principal component analysis.

Transcriptionally, we compared matching PBL and NP samples from sorted PBL nB cells, NP IgD+ B cells, and NP ASC by performing RNA seq analysis (Fig. 8a). There were 1859 differentially expressed genes (DEG) between any pair-wise comparisons with an FDR 0.05. The PBL nB cells and NP ASC clustered separately while the NP IgD+ B cells appeared to be an intermediary phenotype. Interestingly, there were 949 DEG upregulated between NP and PBL nB cells but only 370 DEG between NP IgD+ B cells vs NP ASC (Fig. 8b). Similarly, there were 471 DEG that were downregulated between NP IgD+ & PBL nB cells and only 69 DEG between NP IgD+ B cells & NP ASC.

The PCA plots show that the NP IgD+ B cells are an intermediary phenotype (Fig. 8c). From the RNA seq, the PBL nB cells had increased B cells transcription factors (BACH2 and Pax5) and the NP ASC had increased expression of plasma cell genes (PRDM1 or BLIMP1 and XBP1) (Fig. 8d). The NP IgD+ B cells had notable downregulation of BACH2 and Pax5 and upregulation of PRDM1 and XBP1 compared to PBL nB cells. Manual qPCR further validated these results from three additional subjects (Fig. 8e). Based on a continuum of PBL nB cells to ASC, the intermediate NP IgD+ B cells are closer to ASC based on the DEGs.

In a supervised analysis, we also observed increased expression BACH2, Pax5, SPIB, ETS1, IRF8, CIITA in PBL nB cells and decreased expression of these genes in NP IgD+ B cells and NP ASC (Fig. 8f). Additionally, PBL nB cells had higher expression of IL-4R, CD72, FoxP1, REL, TGFβR2, and FCRL2. In contrast, NP ASC had higher BLIMP1, XBP1, IRF4, SDC1 (CD138), and CD38 compared to NP IgD+ and PBL nB cells. In concordance with the flow data, NP ASC also had higher expression in SLAMF7, as well as increased IL-5Ra, DUSP5, IL-6R, IL-6ST, and CDC25a compared to other cell types (Fig. 8f). As expected, immunoglobulin genes had the highest expression in NP ASC with decreasing levels in NP IgD+ B cells and with the lowest expression in PBL nB cells. IgE expression was notable in only ASC and partially increased in NP IgD+ cells suggesting rapid differentiation to ASC after IgE class switching. We observed differences in the transcript levels of IgE vs other isotypes suggesting that differential expression of IgE transcripts.

## Discussion

In summary, this study demonstrates that local IgD+ B cells are a major source of NP IgE ASC through an EF differentiation pathway compared to other potential sources such as IgG and IgA memory cells. The mucosal IgE ASC have an average lower mutation rates compared to IgG and IgA ASC suggesting origins of the lowly mutated IgM/IgD mucosal IgD+ B cells. These cells were reminiscent of the recently characterized EF activated naive B cells described in autoimmunity and severe SARS-CoV-2 infection. Moreover, both direct and indirect class switching were notable from these cells to IgE ASC. Despite a small fraction of the IgD+ B cells which had higher mutation frequencies, non-canonical switching through IgD to IgE ASC was not evident. These results support the origins of IgD+ mucosal B cells as an important source of mucosal IgE ASC.

NP IgD+ B cells lack expression of CD27 and hence, they display the IgD+CD27- phenotype generally ascribed to naïve B cells^59^. Yet, they display markers of activation and contain a relatively small but significant fraction of isotype switched cells bearing SHM at a significantly lower level than memory cells. The presence of mutated, isotype switched cells within surface IgD+ cells could be the result of contamination during sorting experiments. However, multiple controls argue strongly against such explanation, including the consistent detection by careful analytical flow cytometry of surface IgD+ and intracellular IgG or IgA.

Our results are most consistent with the significant heterogeneity that we and others have identified within the larger parental IgD+CD27- population, a feature more readily documented in SLE patients with active disease and enhanced EF responses^16–18,50,59,60^. We have shown that in such patients, IgD+CD27− cells are highly enriched in an activated fraction that we termed activated naïve B cells (aN). These cells represent the first step of naïve B cell differentiation into extrafollicular ASC through an IgD-CD27− DN intermediate comprised by a large majority of isotype switched cells expressing significant levels of somatic hypermutation albeit of substantially lower magnitude than isotype switched CD27+ memory cells. We have also shown that aN cells readily differentiate into DN cells and that both subsets share an almost identical phenotype and transcriptome. Resonant of SLE aN^18^, the NP IgD+ B cells bear a CD11c+CD86+CD21− phenotype and contain an isotype switched fraction with low but significant levels of SHM. Thus, IgE ASC appear to have similar origins of strong EF differentiation at the local tissue sites from the aN B cells (NP IgD+ B cells). Whether the DN populations are also involved will require additional studies.

With their active participation in an ongoing local, antigen-driven immune response, and unlike resting nB cells in circulation, a plurality of mucosal IgD+ B cells engage the BCR (Nur77+) possibly from aeroallergens/microbial pathogens. Combined with the well-documented evidence in mouse models for isotype switch and SHM in EF responses^5–14,61^ and the recent demonstration that isotype switch occurs mostly outside the germinal center^23^, we postulate that the aggregate of our results strongly suggests that NP IgD+ cells represent a post-naïve activation intermediate that has already initiated isotype switch and somatic hypermutation and is poised to become an ASC in a GC-independent fashion. The importance of this pathway and its applicability to other human immune disorders is emphasized by extensive single cell data from Lupus Nephritis samples recently published by the AMP network^62^. Similarly, the massive ASC in severe patients with SARS-CoV-2 infection also showed evidence of a dominant EF aN B cell response^19^.

Although IgE ASC had lower average mutation rates compared to other switch isotypes, there was a range suggesting that more highly mutated IgE ASC were likely derived from sequential switched IgG B cells and may have higher affinities to aeroallergens and thus result in more prominent IgE-mediated activation of effector cells such as mast cells and basophils.

Additional support of activated B cells is with the similar transcriptomes of NP IgD+ cells and NP ASC in contrast to the transcriptional distance from resting PBL nB cells. Our post-sort purity was 97–99% and even this small contaminating population in the NP IgD+ B cells could not explain that more than 80% of these cells are Nur77 positive and almost 60% are CD86+ and CD71+. Phenotypically, the majority of NP IgD+ are highly activated with as high as 8 and 13% the cells with intracellular IgG or IgA protein despite surface IgD+ staining.

The small but significant fraction of highly mutated NP IgD+ cells demonstrate their heterogeneity. However, it is less likely that they are novel precursors to IgE ASC due to the discrepancy in the mutational analysis. Evidence of the highly mutated IgD ASC have been described with potential roles for basophil activation^52,63–65^. The IgD sequences were also found in the bronchial mucosa of asthmatic individual in higher frequencies compared to healthy controls; however, they could not distinguish B cells from IgD ASC in that study^66^. Our study identified that a small fraction of the IgD sequences are highly mutated from both IgD+CD27− B cells and the IgD ASC by single cell analysis. Although connectivity of this subset with the IgD ASC were limited due to the small numbers of cells, it is quite possible the highly mutated IgD+ B cell subset will be connected to the mucosal IgD ASC.

Our study illustrates a new model of local EF human IgE ASC generation with mucosal IgD+ B cell at the origin. Whether these IgD+ B cells in an allergic patient transit to the mucosa and become activated following engagement by mucosal antigens (microbes or aeroallergens) or are resident B cells is not known. Based on the local cytokine milieu and specific T cells, it appears that some may rapidly class switch and further differentiate into ASC (IgG & IgA) and provide protection at the barrier. In the setting of atopic and some non-atopic airway diseases, the respiratory milieu is skewed to T2 cytokines (such as IL-4, IL-5, and IL-13 from innate lymphoid cells (ILC2s), basophils, and T_H_2 cells) and thus promote active IgE class switching.

IL-4 is critical for IgE class switching and quite surprisingly, we show that PBL nB cells and NP IgD+ B cells have high surface IL-4R expression compared ASC (Figs. 6f and 8f). It is well-known that bystander T cells upregulate IL-4R expression with IL-4/STAT6,^67–69^ but antigen-driven activated T cells have selective downregulation of the IL-4Ra^70^. In all, both PBL nB cells and NP IgD+ B cells appear perfectly poised to differentiate via an IL-4 driven pathway of IgE differentiation.

IL-21 is inhibitory to IgE ASC generation and is an important cytokine secreted by T_FH_ in the GC reactions^27^. IL-21 inhibition of IgE generation further emphasizes the role of EF IgE ASC generation in mucosal sites or EF lymphoid structures.

Our data also support sequential IgE class switching contributes to allergic inflammation. This is evident with clones in class-switched IgG & IgA transcripts in the IgD+ B cells with IgE ASC. Consequently, both direct and sequential IgE class switching from IgD+ B cells may occur in allergic disease.

In the past several years, specific monoclonal antibodies have changed the therapeutic landscape of T2 high diseases such as asthma and atopic dermatitis^71–79^. Omalizumab, an anti-IgE molecule, is efficacious in allergic asthma underlining the functionality of IgE in airway disease. Targets of IL-5 and IL-5 receptorα (IL-5Rα) as well as the IL-4 receptor (IL-4R) have also been shown to be major game-changing approaches to these difficult diseases. Our transcriptomes provide novel mechanistic off target effects of these novel therapies. For example, increased IL-5Rα on mucosal ASC illustrate how ASC (Fig. 8f) may decrease in addition to eosinophils with IL-5 receptor antagonists such as Benralizumab^73–75^. Additionally, IL-4Rα receptors antagonist, Dupilumab, may have unique inhibitory mechanisms on IL-4Rα expressing nB cells. The long-term implications of these immunomodulators will clearly need further studies but our transcriptome analysis provide important clues to how they affect B cells and ASC.

In summary, this study provides support of a novel model of human IgE ASC generation in extrafollicular mucosal sites from IgD+ B cells. Whether the mucosal IgD+ B cells have a persistent reservoir or memory counterpart will need more studies.

## Methods

### Blood & nasal polyp samples from human subjects

Peripheral blood samples were obtained from 32 healthy donors and 83 atopic patients with history of allergic rhinitis and/or asthma based on serum IgE levels >180 IU/mL and positive skin prick testing (serum IgE >0.35 kU/L for common aeroallergens in Georgia) (Supplementary Table 1). Subjects on oral corticosteroids or subcutaneous or sublingual immunotherapy were excluded. Blood samples were analyzed before and/or during the allergy season. The allergy season was defined by periods when the specific aeroallergen counts were high in Atlanta GA, as indicated by the Atlanta, Allergy & Asthma Pollen Counting Stations certified by the National Allergy Bureau (http://www.atlantaallergy.com/). Nearly all patients were symptomatic during the high pollen counts. For this study, 56 NP with matching blood samples were collected during routine endoscopic sinus surgery from patients with Chronic rhinosinusitis (CRS), allergic fungal sinusitis (AFS) or aspirin exacerbated respiratory disease (AERD). Detailed phenotypes of the NP are also shown in Supplementary Table 1. The subjects were enrolled in this study at Emory University between 2014 and 2019. All studies were approved by the Emory Institutional Review Board.

### Cell isolation

Mononuclear cells were isolated from PBL by ficoll density gradient centrifugation as previously described^35^. NP samples were minced using a scalpel, placed in a 50 ml tube with 8 ml DMEM + Liberase TL 125 ug/mL (Roche) + DNAse I 100 ug/ml (SIGMA) + antibiotic/antimycotic (GIBCO), incubated at 37 °C for 45 min and vortexed at low speed each 10 min. After the incubation the suspension was filtered through a sterile 70 um mesh.

### Multi-color flow cytometry and sorting

Mononuclear cells from PBL or NP cells were incubated with normal mouse serum for 20 min, in order to saturate and block nonspecific binding sites, then stained with different combinations of the following anti-human antibodies directed against human molecules: CD3-PE-Cy5.5, CD14-PE-Cy5.5 (Invitrogen, Camarillo, CA), or CD3 BV711,CD14 BV711, CD27 APC- Alexa750, CD20 PE, CD23 Pe-Cy7, CXCR4-PE-Cy5, CD80 PE-Cy7, HLA-DR PE, CD124 PE, intracellular Ki-67-FITC, IRF4-PE, (eBioscience, BioLegend, San Diego, CA), CD19-PE-Cy7 or APC CD11C PE, IgD FITC, CD38-Pacific Blue, HLA-DR-AlexaFluor700, CD21 PE-Cy7, IgG PE or APC, IgA PE or APC, CD71 PE, CD86 PE, IgM PE or APC (BD Pharmingen, San Diego, CA), CD138-APC (Miltenyi Biotec, Aubrun, CA.). Compensation beads (Ultra comp eBeads eBioscience) were used for single stained controls. The cells were analyzed on a BD Biosciences LSRII.

B cells subsets and ASC were sorted with a FACSAria II (BD Bioscience) using the following antibodies: IgD-FITC, CD19-PE-Cy7, CD38-Pacific Blue (BD Pharmingen, San Diego, CA), CD3-PE-Cy5.5, CD14-PE-Cy5.5 (Invitrogen, Camarillo, CA) or CD3 BV711, CD14 BV711, (eBioscience, BioLegend, San Diego, CA), CD138-APC (Miltenyi Biotec, Aubrun, CA), and CD27 APC- Alexa750 (eBioscience, San Diego CA). Approximately 0.1 to 5 × 10^5^ cells were collected for each subset.

*ASC Elispots* were performed as previously described^35,80,81^. Briefly, PBMC, nasal polyp cells or sorted B cell subsets were added to 96-well ELISpot plates (MAIPS4510 96 well) previously coated with anti-human IgG and anti-human IgA 5 μg/mL (Jackson Immunoresearch, West Grove, PA), anti-human IgM 5 μg/mL (Invitrogen) anti-human IgE 5 µg/ml, (MabTech), anti-human IgG_4_ 10 µg/ml (BD), and incubated overnight. Wells were washed thoroughly with water containing 1× PBS and 0.1% Tween 20 (PBST), followed by incubation for two hours at room temperature (RT) with 1 µg/ml Alkaline phosphatase (ALP)-conjugated goat anti-human IgG antibody, 1 µg/ml ALP-conjugated, goat anti-human IgA, 1 µg/ml alkaline phosphatase-conjugated goat anti-human IgM, biotinylated mouse anti-human IgE, Mabtech. For the IgG_4_ was used 0.5 µg/ml of alkaline phosphatase (ALP)-conjugated goat anti-human IgG antibody, (Jackson Immunoresearch). All the secondary antibodies were diluted in PBST with 2% BSA (PBSTB). After washing, 50 uL of streptavidin-AP (1 µg/ml) to IgE wells was added. The plate was incubated for 1 h at room temperature. Plates were then washed and developed with Vector AP substrate kit III solution (for IgG, IgA, IgM and IgG_4_) or NBT/BCIP, Mabtech, solution (for IgE) at RT. Spots in each well were counted using the CTL Immunospot reader (Cellular Technologies Ltd).

We considered only the darker spots as IgE produced by the ASC and hypothesized that the smaller spots in the background were due to IgE released after nonspecifically binding to CD23 on B cells. Treating the PBMC with the acid buffer to release the IgE bound to CD23^82,83^, we observed that only the darker spots corresponded to the IgE actually produced by the ASC (data not shown).

### IgE ELISA

Elisa assay was performed using the human IgE ready set to go kit (Affimetrix ebioscience) following the manufacturer’s protocol.

### Quantitative PCR

RNA from sorted cells was isolated using the RNeasy Mini Kit (Qiagen Inc., Valencia, CA) by following the manufacturer’s protocol. Approximately 400 pg of RNA was subjected to reverse transcription using the iScript RT Kit (BioRad Inc., Hercules, CA). qRT-PCR was performed using iQ SYBR Green supermix (BioRad Inc. Hercules, CA) following the manufacturer’s protocol. All samples were run in triplicates. Resulting CT values were normalized to β actin and expressed as 2-dct. Table of QRT-PCR primers listed (forward 5′->3′, reverse 5′->3′):

εGLT: (CTGTCCAGGAACCCGACAGA,TGCAGCAGCGGGTCAAG)

XBP1: (CTGAGTCCGCAGCAGGTG, GGCTGGTAAGGAACTGGGTC)

PAX 5: (GAGCGGGTGTGTGACAATGA, GCACCGGAGACTCCTGAATAC)

BACH 2: (GCGGAAAAGGACGCAAAGTT, AAGGGCTCATCAGCTTGGTC)

BLIMP 1: (AACTTCTTGTGTGGTATTGTCGG, TCTCAGTGCTCGGTTGCTTT)

β-actin: (CTGGAACGGTGAAGGTGACA, AAGGGACTTCCTGTAACAATGCA)

### Cytospins of sorted B Cell subsets

Sorted cells from PBMCs and NP samples were spun at 1300 RPM for 5 min using the Cytospin 4 (Thermo Scientific, Waltham, MA). Approximately 5000 cells per subset were dried on albumin coated slides and colored with Wright-Geimsa stain.

### Library construction and NGS of the IGH repertoire

Approximately 2 ng of RNA isolated from sorted cells using the RNeasy micro kit (Qiagen) following the manufacturer protocol, was subject to reverse transcription with an iScript cDNA synthesis kit (Biorad). Initial PCR was carried out using isotype-specific sequences as reverse primers targeting the region encoding the first constant domain of each isotype Cα, Cμ, Cγ and Cε (250 nM) in combination with a mix of VH1- VH7 framework region 1 as forward primer (50 nM), preceded by the respective Illumina Nextera sequencing tag in a PCR volume of 25μl using the high-fidelity Platinum PCR Supermix (Invitrogen). Each isotype was amplified individually, in order to minimize the risk of PCR crossover errors and chimeric PCR artifacts involving sequences encoding different antibody isotypes. Amplification was performed with a BioRad C1000 thermal cycler in the following conditions: 3 min at 95 °C, 40 Cycles of: 30 s at 95 °C, 30 s at 58 °C, 30 s at 72 °C, 5 min at 72 °C and hold at 10 °C. Nextera indices (Illumina, San Diego, CA) carrying sequencing adapters optimized for MiSeq sequencing were added using a BIO-TAD C1000 thermal cycler with the following conditions: 30 s at 98 °C, 5 Cycles of: 10 s at 98 °C, 30 s at 63 °C, 3 min at 72 °C. PCR products were purified using Agencourt AMPure XP beads (Beckman Coulter, Brea, CA) quantified using the pico-green (Quant-iT™ PicoGreen™ dsDNA Assay Kit) and finally pooled and denaturated. Single strand sequencing was performed using MiSeq technology (Illumina) with a 2 × 301 setup in High Output mode.

Specifically, we performed separate RT-PCR for each Ig isotype to avoid cross-isotype chimeric PCR product formation and pooled equal amounts of PCR product for each isotype. This method increases accuracy but would not allow isotype quantification for each population. Individual clones were defined if they shared rearrangements of V_H_ and joining heavy-chain (JH) segments, identical immunoglobulin heavy chain complementarity determining region 3 (HCDR3) length, and a Hammig identity of >85% for HCDR3 as previously described^18^.

Primers used for NGS (forward 5′->3′, reverse 5′->3′)

Forward:

VH1:GGCCTCAGTGAAGGTCTCCTGCAAG

VH2:GTCTGGTCCTACGCTGGTGAAACCC

VH3:GGTCCCTGAGACTCTCCTGT

VH4:ACCCTGTCCCTCACCTGC

VH5:GCAGCTGGTGCAGTCTGGAG

VH6:CAGGACTGGTGAAGCCCTCG

VH7:CAGGTGCAGCTGGTGCAA

Reverse:

Cm:CAGGAGACGAGGGGGAAAAGG

Cg:CCGATGGGCCCTTGGTGGA

Ca:GAAGACCTTGGGGCTGGTCG

Ce: TTGCAGCAGCGGGTCAAGGG

F tag: 5′-TCGTCGGCAGCGTCAGATGTGTATAAGAGACAG-3′

R tag: 5′-GTCTCGTGGGCTCGGAGATGTGTATAAGAGACAG-3′

### 10× genomics single cell VDJ sequencing

Single cell encapsulation and subsequent first strand cDNA synthesis were carried out via manufacturer’s protocols on a 10× Genomics Chromium platform. Target enrichment of VDJ transcripts was also performed according to manufacturer’s protocols (Chromium Single Cell V(D)J Enrichment Kit, Human B cell; CG000166 Rev A) and using a BioRad C1000 thermal cycler. Products were then sequenced on an Illumina MiSeq instrument using a v3 150-cycle reagent kit at a read depth of ~5k reads per cell. Data was deconvoluted, aligned to consensus reads, and clustered into clones using Cell Ranger software (v3.0.2) in basic operation using a Linux workstation. Additional mucosal ASC were sorted for single cell encapsulation and first strand cDNA synthesis were performed as above according to manufacturer’s protocol. cDNA was sequenced to a depth 20,000 reads per cells and aligned using Cell Ranger software. Sequences were aligned in IMGT and analysis performed.

### Bioinformatic analysis of NGS data

The analysis of the sequencing data was carried out using an informatic pipeline developed in-house. The first step consists of joining paired end reads and then filtering the sequences on the basis of length and quality threshold. Sequences smaller than 200 bp were excluded from the analysis. Isotype were identified through alignment of the constant region segment of each sequence. Quality filtered repertoire sequences were clustered into B cell lineages as previously described^23,24^. Briefly, B cell clones were defined as those sequences sharing identical V and J selection, identical CDR3 length, and at least 85% sequence homology and assigned a unique lineage identifier. The percentage of lineages shared between subpopulations (i.e., NP naive, PBL ASC, etc.) was used as a measure of population relatedness. Circular visualization plots were created using circos v0.64 (http://circos.ca/).

### RNA seq methods

1–10 ng of total RNA was used as input for the mRNA HyperPrep Kit (Kapa Biosystems) according to the manufacturer’s instructions. Following adapter ligation cDNA was PCR amplified for 12 cycles. Final libraries were QC’ed on a bioanalzyer, quantitated by qPCR, and pooled at equimolar ratios for sequencing on a NextSeq500 using 75 bp paired-end chemistry.

### RNA seq analysis

Methods: Reads were mapped to hg38, using STAR (https://www.ncbi.nlm.nih.gov/pmc/articles/PMC3530905/) to generate bam files. The mapped libraries were then normalized accounting for differences in size and dispersion using the default parameters of EdgeR for gene abundance levels. Read counts were converted to the log base 2 scale and principal component variance analysis (PCVA) in JMP-Genomics (version 8.0) was used to assess the contributions of Individual, Batch, Cell Population, and for differential expression analysis. We set a lower threshold of 3 log2 units, selected by plotting the coefficient of variance against average abundance. Lower-abundance features were removed for all downstream analyses. Differences among cell populations were assessed by analysis of variance on a gene-by-gene basis. Principal component analysis was also performed on the 1257 differentially expressed genes. Gene set enrichment analysis (GSEA) was performed using the t-statistic (derived from comparison of each gene between each pair of populations) to rank genes, and pre-ranked gene set sets significantly enriched for high or low expression with FDR < 0.05 were deemed to be dysregulated between populations. The expression of the standardized least-square-means (SLSM) for each gene in each gene set was then used to compute PC1 for the gene set, and these values were hierarchically clustered again using Ward’s method in JMP Genomics.

### Immunohistochemistry and immunofluorescent staining

Immunohistochemical staining on sections of NP was performed using a biotin-free polymer system. The paraffin-embedded sections were subjected to deparaffinization in xylene, rehydration in graded series of ethanol, and rinsed with double distilled water. Antigen retrieval was performed by immersing sections in DIVA Decloaker (Biocare Medical) at 125 °C for 30 s in a steam pressure decloaking chamber (Biocare Medical) followed by blocking with SNIPER Reagent (Biocare Medical) for 10 min. The sections were incubated with mouse anti-human CD20 (clone L26; Dako) or rabbit anti-human CD3 (polyclonal Ab; Dako) for overnight at 4 °C followed by a detection polymer system (MACH 2; Biocare Medical). Antibody labeling was visualized by development of the chromogen (Warp Red Chromogen Kit; Biocare Medical). Digital images of stained slides were captured at 40×, 400× and 600× magnification with an Olympus BX43 microscope equipped with a digital camera (DP26, Olympus).

Similarly, Immunofluorescence staining was performed using a modified protocol previously described^84^. Sections were incubated with optimized concentrations of antibodies as described above overnight. Thereafter, the sections were washed and incubated with conjugated secondary Abs (Alexa Fluor 488/555, Abcam) at RT for 1 hour. Following incubations, the slides were washed twice with TBS buffer. Upon completion of immunofluorescence staining, the sections were mounted with ProLong Gold anti-fade reagent with DAPI (4′,6-diamidino-2-phenylindole) (Life Technologies) as a nuclear counter-stain and coverslipped. Images were captured using Olympus IX83 fluorescence microscope.

The bulk VDJ repertoire data are available from the NCBI database under accession PRJNA728136. The single cell VDJ data are available from the NCBI database under accession GSE174349. The bulk RNA-seq data are available from the NCBI database under accession GSE174681.

## Supplementary Information

Supplementary Information
