# Supplementary material for: Extrafollicular IgD+ B cells generate IgE antibody secreting cells in the nasal mucosa
Source: Mucosal Immunol. 2021 May 28;14(5):1144–59. doi: 10.1038/s41385-021-00410-w (PMC8160425; doi:10.1038/s41385-021-00410-w)
Supplement: Supplementary file 1 — Supplementary Information [file 41385_2021_410_MOESM1_ESM.pdf]

Supplementary figure 1

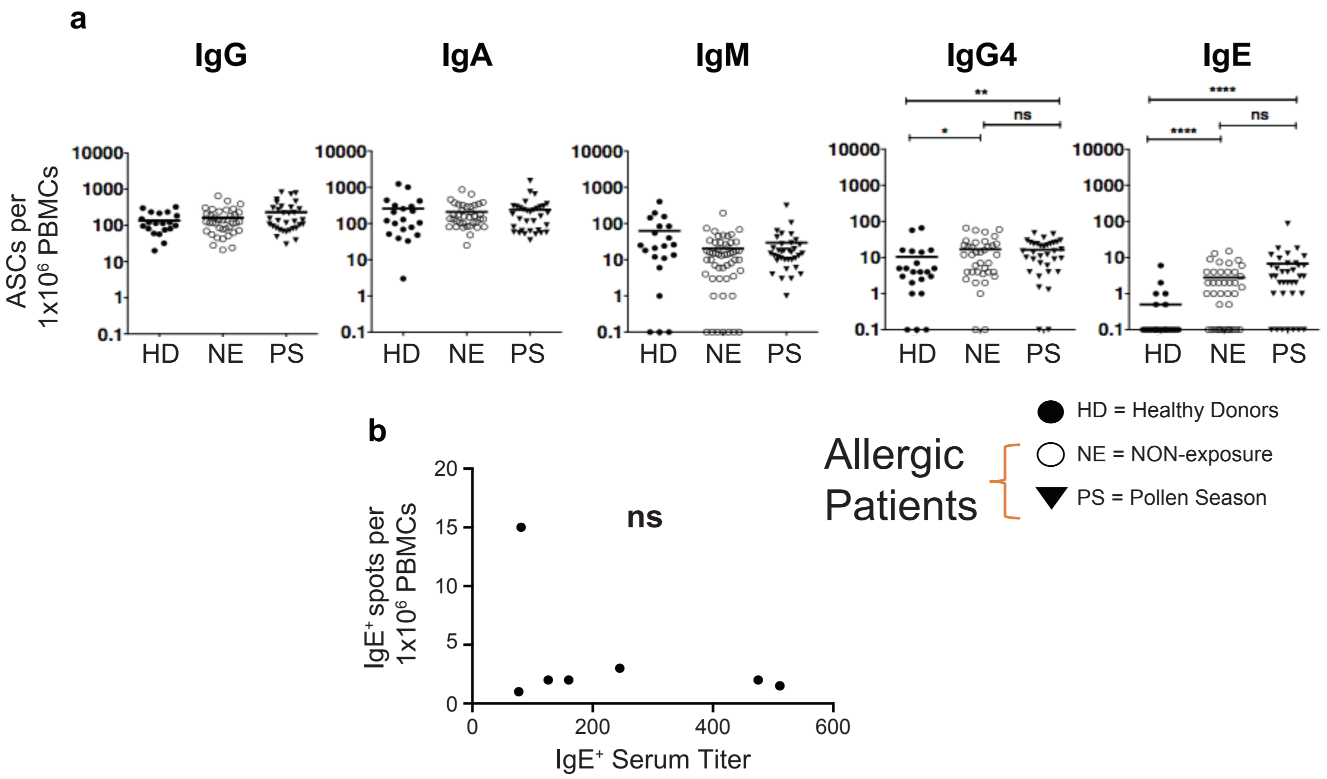

Supplementary figure (1a). Frequencies of IgG4 and IgE ASC Elispots in PBMC from non-allergic healthy donors (HD) and patients with high serum IgE levels (>180 IU/mL). Blood samples analyzed prior to the allergy season (non-exposure: NE) and during the pollen season (PS). Mean value indicated in each graph. Mann Whitney test (two tailed) (p-value * 0.05, ** 0.01, *** 0.001, **** 0.0001. (1b) Lack of correlation of IgE ASC with serum IgE levels.

Supplementary figure 2

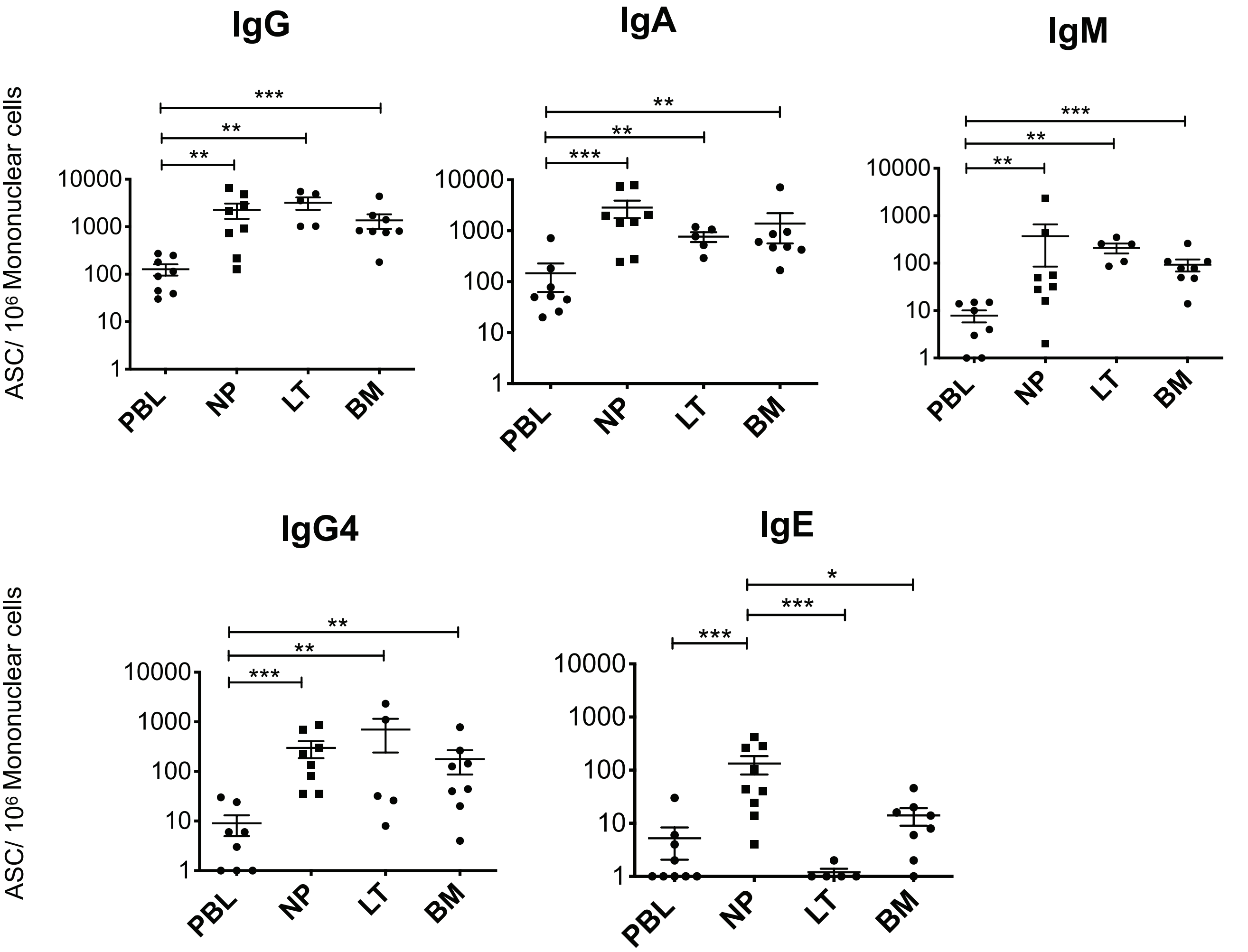

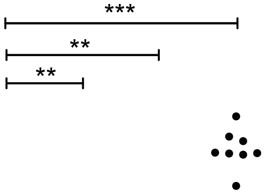

Supplementary figure 2. Frequencies of IgG, IgA, IgM, IgG_4, and IgE ASC Elispots from 9 matching NP and PBL sample, 5 separate lymphoid tissues LT (adenoids and tonsils) (non-matching samples), and BM from 8 atopic adults. ASC Elispots (IgG, IgA, IgM, IgG4, & IgE) Mean + SEM: (Mann-Whitney, p-value * 0.05, ** 0.01, *** 0.001).

Supplementary figure 3

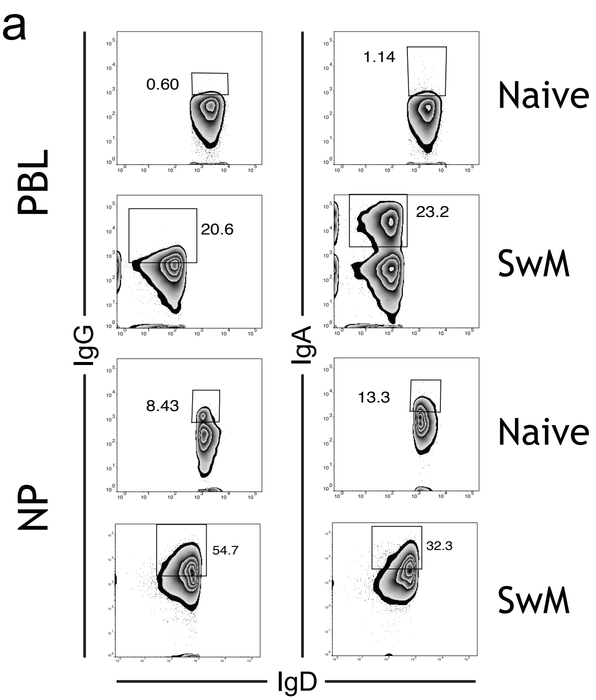

b

Percentage of each isotype subclass

PBL Naive		NP Naive		NP ASC	
24.56	IGHD	3.93	IGHA1	26.27	IGHA1
75.44	IGHM	2.62	IGHA2	5.07	IGHA2
		19.21	IGHD	5.53	IGHE
		1.75	IGHG1	13.82	IGHG1
		2.18	IGHG2	21.66	IGHG2
		70.31	IGHM	1.84	IGHG3
				0.46	IGHG4
				25.35	IGHM

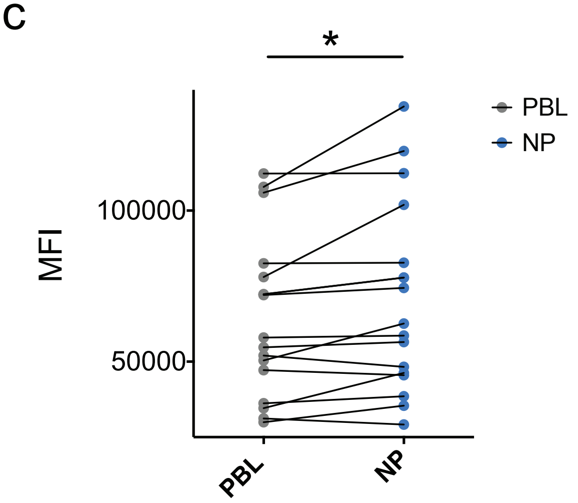

Supplementary figure 3. (a) Representative surface staining of Naïve and SwM B cells from blood and matching nasal polyp. Starting from the right column: IgG vs IgD, IgA vs IgD and IgM vs IgD. (b) Percentages of each isotype subclass express in FAC sorted PBL Naïve, NP Naïve and NP ASC of one nasal polyp and matching blood sample, obtained with 10X technology. (c) Geometric Mean of Forward scatter fluorescent intensity of matching blood naive (gray) and nasal polyp IgD+ B cells (blue, (paired t test).

Supplementary table 1

Baseline Demographic Characteristics

Characteristic	Atopic Patients (n = 83)	Healthy Adults (n = 32)	Nasal Polyps Patients (n = 54)
Gender – no. (%)			
Male	21 (25.3%)	12 (37.5%)	31 (57.4%)
Female	62 (74.7%)	20 (62.5%)	22 (40.7%)
Unknown			1 (1.8%)
Age – yr	51±16.5	33±10.5	44.1±15
Race – no. (%)			
African American	48 (57.8%)	7 (21.8%)	28 (51.8%)
Caucasian	30 (36.1%)	20 (62.5%)	24 (44.4%)
Asian	5 (6%)	5 (15.6%)	0 (0%)
Unknown			2 (3.7%)
Serum IgE – IU/mL	633.78 ± 619		273.2±127
Diagnosis – no. (%)			
CRSwNP			34 (62.9%)
AFS			15 (27.7%)
AERD			4 (7.4%)
Unknown			1 (1.8%)

*Values include mean ± standard deviation

Supplementary table 2

Patient ID	Population	Sorted Cells	Sequences	Lineages
**674**	***np_asc***	35949	1715363	4643
	np_IgD+	10000	425519	2793
	np_CD27+M	3159	230394	989
	pbl_asc	556	433713	321
	pbl_naive	10000	167870	1432
	pbl_CD27+M	30000	437843	1383
**1095**	***np_asc***	55380	661451	3837
	np_IgD+	22053	87172	228
	pbl_asc	16696	266684	7572
	pbl_naive	346352	78878	45703
	pbl_CD27+M	80000	132568	14963
**1325**	***np_asc***	17120	397635	2351
	np_IgD+	3660	134133	1122
	pbl_asc	36700	265547	10672
	pbl_naive	200000	130276	25798
	pbl_CD27+	200000	132789	15112
**1417**	***np_asc***	42648	502185	3764
	np_IgD+	31050	129122	4271
	np_CD27+	10100	128539	1052
	pbl_asc	11163	263934	4267
	pbl_naive	153821	85403	25457
	pbl_CD27+	50569	59755	6298
**1789**	***np_asc***	66100	350294	5171
	np_IgD+	10900	72503	820
	np_CD27+M	16100	24220	1234
	pbl_asc	9700	153533	4467
	pbl_naive	100000	133870	21835
	pbl_CD27+	100000	214123	21031
**1792**	***np_asc***	30104	434313	2229
	np_IgD+	7460	369988	1300
	np_CD27+M	7648	238	50
	pbl_asc	3397	25960	271
	pbl_naive	60000	116445	1300
	pbl_CD27+M	58760	426945	18666
**1809**	***np_asc***	228000	475650	2997
	np_IgD+	18000	101024	657
	np_CD27+M	15400	57151	869
	pbl_asc	9100	98304	1856
	pbl_naive	602600	66637	11301
	pbl-CD27+M	123700	40318	1107

Supplementary Table 3

	IRF4	CD 38++	SLAM 7	Ki67	CD20	CD21
	N=2	N=13	N=2	N=5	N=1	N=6
**NP Naïve % positive**	**14**	**7**	**7**	**22**	**70**	**40**
Range	(4-24)	(1-16)	(6-8)	(9-44)		(12-79)
**PBL Naive % positive**	**2**	**2**	**1**	**4**	**100**	**90**
Range	(2.3-2.4)	(0-8)		(1-12)		(82-96)
**NP ASC % Positive**	**98**	**100**	**99**	**65**	**8**	**30**
Range	98		(98-100)	(46-79)		(15-50)
**PBL ASC % Positive**	**97**	**100**	**88**	**87**	**26**	**23**
Range	(95-99)		(86-91)	(71-99)		(17-44)

	CD23	CD80	CD86	CD71	CD11C	A4B7
	N=4	N=3	N=3	N=3	N=6	N=3
**NP Naïve % positive**	**34**	**8**	**40**	**40**	**16**	**48**
Range	(15-57)	(6-10)	(30-59)	(26-64)	(2-43)	(44-53)
**PBL Naive % positive**	**80**	**4**	**2**	**2**	**5**	**84**
Range	(63-96)	(3-5)	(1-3)	(1-5)	(2-9)	(82-86)
**NP ASC % Positive**	**21**	**15**	**72**	**55**	**41**	**74**
Range	(4-41)	(5-31)	(64-93)	(35-77)	(35-47)	(56-92)
**PBL ASC % Positive**	**7**	**16**	**89**	**24**	**19**	**43**
Range	(2-14)	(6-26)	(75-96)	(9-46)	(16-23)	(25-61)

	CXCR4	Surface IgG	SurfaceIgA
	N=5	N=7	N=4
**NP Naïve % positive**	**61**	**10**	**16**
Range	(21-94)	(2-41)	(5-26)
**PBL Naive % positive**	**85**	**1**	**1**
Range	(73-98)	(0-2)	(0-3)
**NP ASC % Positive**	**36**	**23**	**45**
Range	(10-51)	(2-54)	(29-61)
**PBL ASC % Positive**	**24**	**10**	**33**
Range	(3-77)	(3-16)	(23-44)
